# Neuroprotective and Symptomatic Effects of Cannabidiol in an Animal Model of Parkinson’s Disease

**DOI:** 10.3390/ijms22168920

**Published:** 2021-08-18

**Authors:** Claudio Giuliano, Miriam Francavilla, Gerardo Ongari, Alessandro Petese, Cristina Ghezzi, Nora Rossini, Fabio Blandini, Silvia Cerri

**Affiliations:** 1 Unit of Cellular and Molecular Neurobiology, IRCCS Mondino Foundation, 27100 Pavia, Italy; claudio.giuliano@mondino.it (C.G.); miriam.francavilla@mondino.it (M.F.); gerardo.ongari@mondino.it (G.O.); alessandro.petese01@gmail.com (A.P.); cristina.ghezzi@mondino.it (C.G.); silvia.cerri@mondino.it (S.C.); 2 Linnea SA, CH-6595 Riazzino, TI (CH), Switzerland; nrossini@linnea.ch; 3 Department of Brain and Behavioral Sciences, University of Pavia, 27100 Pavia, Italy

**Keywords:** phytocannabinoids, neuroinflammation, ciliary neurotrophic factor, transient receptor potential vanilloid 1, 6-hydroxydopamine, cannabidiol

## Abstract

Parkinson’s disease (PD) is a neurodegenerative disorder characterized by the loss of dopaminergic neurons in the Substantia Nigra pars compacta, leading to classical PD motor symptoms. Current therapies are purely symptomatic and do not modify disease progression. Cannabidiol (CBD), one of the main phytocannabinoids identified in Cannabis Sativa, which exhibits a large spectrum of therapeutic properties, including anti-inflammatory and antioxidant effects, suggesting its potential as disease-modifying agent for PD. The aim of this study was to evaluate the effects of chronic treatment with CBD (10 mg/kg, i.p.) on PD-associated neurodegenerative and neuroinflammatory processes, and motor deficits in the 6-hydroxydopamine model. Moreover, we investigated the potential mechanisms by which CBD exerted its effects in this model. CBD-treated animals showed a reduction of nigrostriatal degeneration accompanied by a damping of the neuroinflammatory response and an improvement of motor performance. In particular, CBD exhibits a preferential action on astrocytes and activates the astrocytic transient receptor potential vanilloid 1 (TRPV1), thus, enhancing the endogenous neuroprotective response of ciliary neurotrophic factor (CNTF). These results overall support the potential therapeutic utility of CBD in PD, as both neuroprotective and symptomatic agent.

## 1. Introduction

Parkinson’s disease (PD) is a progressive movement disorder and its prevalence in the population is rapidly rising, with a consequent increasing economic burden on society, in terms of medical care. Indeed, the number of individuals affected by PD was estimated to have more than doubled globally from 1990 to 2016 [1]. Several efforts are currently ongoing to address this health challenge, including the attempt to develop new therapeutic strategies that can modify the course of PD, by modulating the underlying pathogenetic mechanisms such as the inflammatory process [2,3]. Although it is widely recognized that glial cell activation can contribute to the progression of neuronal damage in PD [2,4], it is interesting to note that microglia and astrocytes can polarize into pro- and anti-inflammatory phenotypes in response to changes in brain microenvironment [3,4,5].

Phytochemicals offer a wide variety of compounds with potential therapeutic uses in PD, due to their anti-inflammatory and antioxidant properties. In particular, phytocannabinoids have shown both neuroprotective capacity and the potential to alleviate motor symptoms [6], which makes them an attractive class of compounds to evaluate as potential innovative therapeutics for PD. Their effects have been extensively studied over the years in various neurodegenerative pathologies, such as Huntington’s disease [7,8], Alzheimer’s disease [9,10], Multiple Sclerosis [11,12] and Amyotrophic Lateral Sclerosis [13], yielding promising results.

Cannabidiol (CBD) is one of over 100 phytocannabinoids identified in Cannabis Sativa and constitutes up to 40% of the plant extract, being the second most abundant component. Unlike Δ9-tetrahydrocannabinol, which combines therapeutic properties with some important adverse effects, CBD is not psychoactive [14] and exhibits a large spectrum of therapeutic properties among which the anti-inflammatory and antioxidant effects emerge [15,16,17,18]. These properties make CBD a therapeutic agent potential capable of counteracting the progression of nigrostriatal damage that characterizes PD.

The aim of this study is to evaluate the effects of chronic treatment with CBD on neurodegenerative and neuroinflammatory processes, and motor deficits in a classic toxic model of PD based on unilateral intrastriatal injection of 6-hydroxydopamine (6-OHDA). The potential mechanisms by which CBD exerted its effects in this model have been also investigated. CBD-treated animals showed a reduction of nigrostriatal degeneration accompanied by a recovery of motor performance. In particular, CBD, by activating the astrocytic transient receptor potential vanilloid 1 (TRPV1) [19], enhanced the endogenous neuroprotective response of ciliary neurotrophic factor (CNTF). Specifically, activation of TRPV1 on astrocytes increases endogenous CNTF synthesis in vivo, enhancing the viability of dopaminergic neurons through activation of the CNTF receptor alpha subunit (CNTFRα) and preventing neurodegeneration and inducing the recovery of motor performance after administration of MPP^+^ and 6-OHDA in PD rat models [20,21,22,23].

## 2. Results

### 2.1. Cannabidiol Treatment Attenuates Nigrostriatal Degeneration and Improves Motor Performance

**Figure 1:**
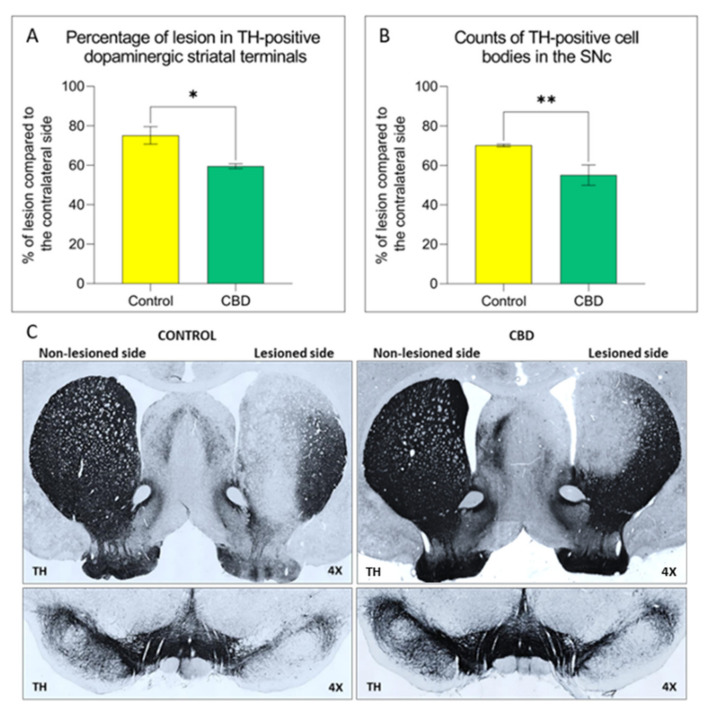
Effect of CBD treatment on the neurodegenerative process in the nigrostriatal pathway. The graphs show the percentage of lesion of (**A**) in the striatum. (**B**) in the SNc. (**C**) Representative images of the nigrostriatal damage in both experimental groups. Results are expressed as mean ± SEM. * *p* < 0.05 vs. control t = 2947, df = 12. ** *p* < 0.01 vs. control t = 3102, df = 13, Student’s *t*-test unpaired two-tailed. N = 6 to 8 in each group.

**Figure 2:**
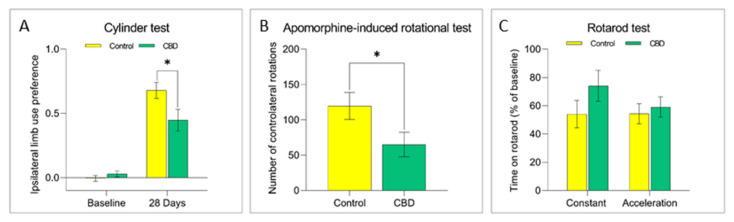
Effects of chronic treatment with CBD on motor behavior of 6-OHDA lesioned rats. The graphs show the motor performance evaluated using the (**A**) Cylinder, (**B**) Apomorphine-induced rotational and (**C**) Rotarod test in both experimental groups. Results are expressed as mean ± SEM. * *p* < 0.05 vs. control t = 2218, df = 39 (Cylinder), t = 2109, df = 28 (Apomorphine), Student’s *t*-test unpaired two-tailed. N = 9 to 20 in each group.

The potential effects of CBD treatment on PD progression were investigated by using the unilateral intrastriatal 6-OHDA-lesion model. The 6-OHDA injection in the striatum causes a partial and gradual degeneration of the nigrostriatal pathway, providing a therapeutic window (28 days) useful to evaluate the neuroprotective efficacy of new therapeutic strategies [24]. In this study, the 6-OHDA injection induced a 75% loss of dopaminergic striatal terminal and 70% loss of dopaminergic neurons in the SNc after 28 days (Figure 1). Animals treated with CBD showed a 21% significant reduction of the striatal terminal degeneration (Figure 1A; *p* = 0.012) and cell body loss in the SNc (Figure 1B; *p* = 0.008) in comparison with control group. CBD also ameliorated 6-OHDA-induced motor deficits as assessed by behavioral tests. Lesioned animals showed a prevalent use of the forepaw ipsilateral to the lesioned side over the contralateral, “parkinsonized” forepaw at cylinder test. Interestingly, animals treated with CBD displayed a significantly lower preference (34%; *p* = 0.032) in the use of the ipsilateral limb than control rats, indicative of a behavioral rescue (Figure 2A). Analogously the apomorphine-induced rotational behavior, a functional index of nigrostriatal lesion in 6-OHDA animal models, was significantly reduced (54%) (*p* = 0.044) in the animals treated with CBD (Figure 2B). Last, the rotarod test was performed to evaluate the motor coordination of rodents. While the analysis of baseline (pre-lesion) motor activity did not reveal any difficulty to stay in balance on the rotating bar in both experimental groups, after nigrostriatal lesion, animals exhibit a reduction (about 50%) in the motor performance as shown in the control group. CBD treatment did not ameliorate motor performance at this test (Figure 2C).

### 2.2. Cannabidiol Modulates the Neuroinflammatory Process in the SNc through a Preferential Action on Astrocytes

**Figure 3:**
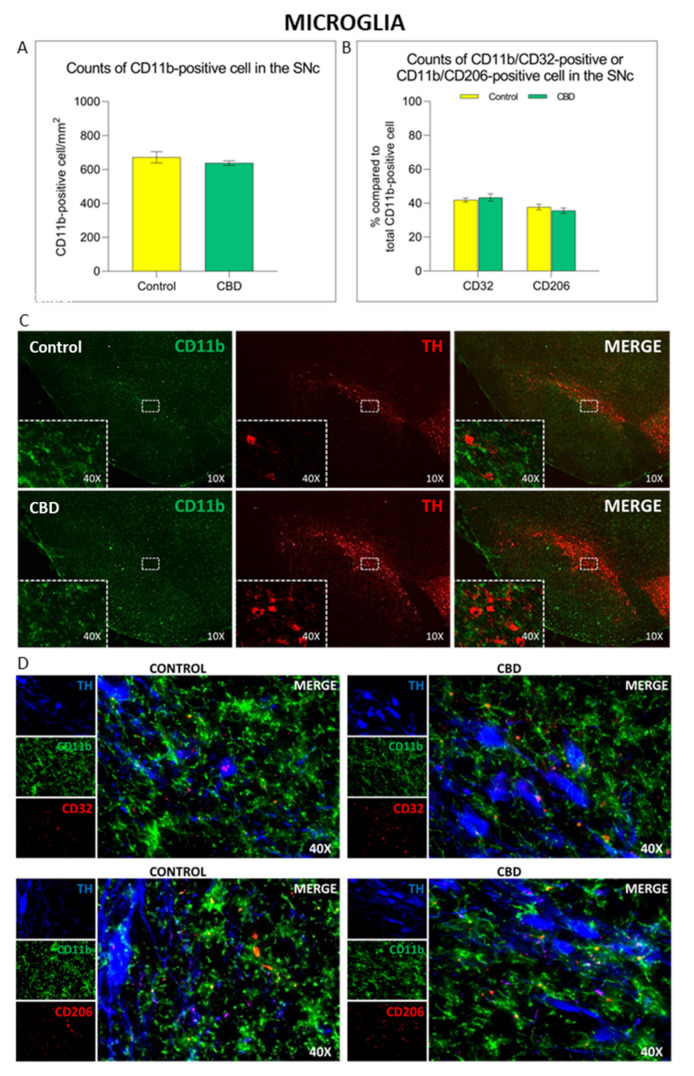
Immunomodulatory effect of chronic treatment with CBD on microglia activation and polarization in the SNc. Com-parison of (**A**) CD11b^+^ cell density and (**B**) microglia polarization state in the SNc in both experimental groups. (**C**,**D**) Representative images of the microglia activation and polarization in the SNc in both experimental groups. The results are expressed as mean ± SEM. N = 6 to 8 in each group.

**Figure 4:**
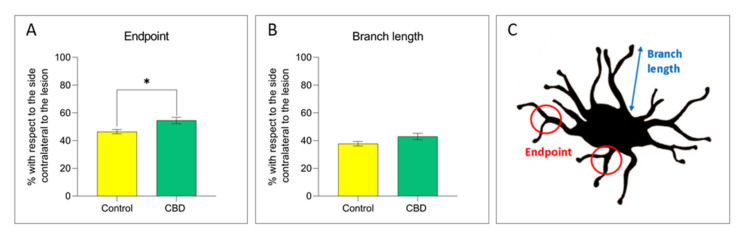
The graphs show the percentage of the (**A**) number of endpoints/cell and (**B**) branch length/cell (µm) in the SNc lesioned side with respect to the unlesioned side in both experimental groups. (**C**) Parameters analyzed to study microglial morphology. Results are expressed as mean ± SEM. * *p* < 0.05 vs. control t = 2992, df = 13, Student’s *t*-test unpaired two-tailed. N = 5 to 8 in each group.

The 6-OHDA-induced neurodegenerative process is accompanied by a neuroinflammatory response in the SNc characterized by the increase in glial cells density (microglia and astrocytes) and a change towards a pro-inflammatory phenotype [25]. No differences in the number of CD11b^+^ cells/mm [2] and in microglia cell polarization towards the cytotoxic M1 (CD11b^+^/CD32^+^) or cytoprotective M2 phenotype (CD11b^+^/CD206^+^) were observed in animals treated with CBD compared to controls, as shown in Figure 3. As we did not observe any effects on microglia density and phenotype, potential changes in microglia morphology after CBD treatment have been investigated. CBD-treated animals showed an 8% increase in the number of microglia endpoints (*p* = 0.010) (Figure 4A,C), accompanied by an increment in the length of microglia branches (Figure 4B,C), but the effect was not statistically significant (*p* = 0.082).

**Figure 5:**
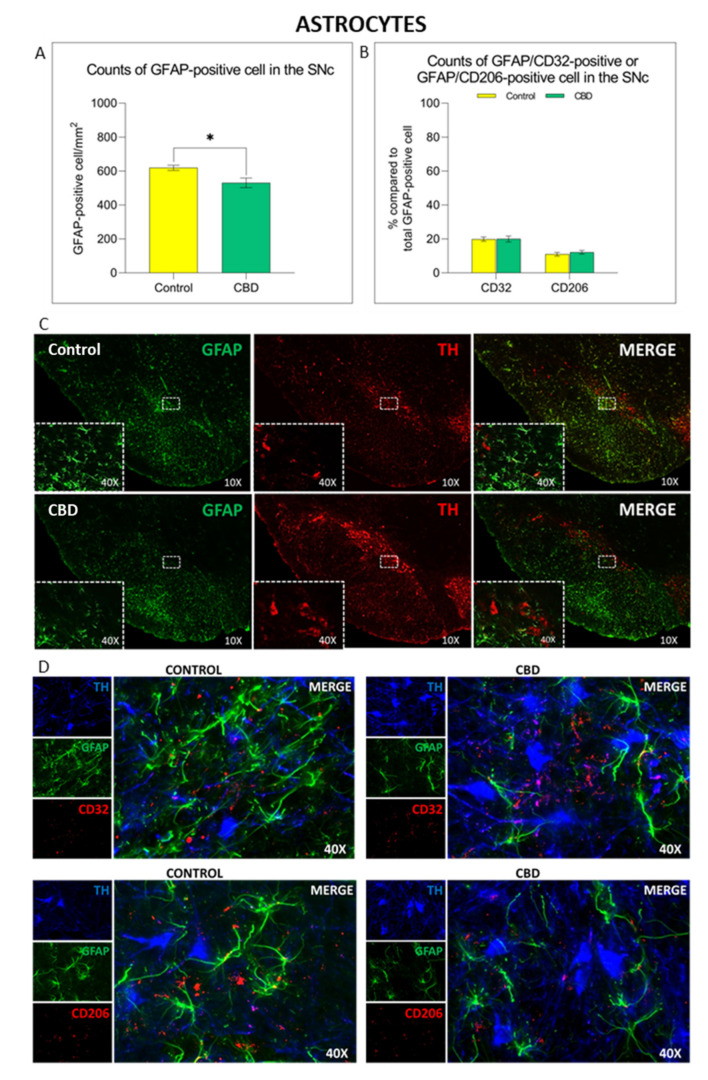
Immunomodulatory effect of chronic treatment with CBD on astrocytes activation and polarization in the SNc. (**A**) Comparison of GFAP^+^ cell density in the SNc in both experimental groups. (**B**) Comparison of astrocyte polarization in SNc in both experimental groups. (**C**,**D**) Representative images of the astrocyte activation and polarization in the SNc in both experimental groups. Results are expressed as mean ± SEM. * *p* < 0.05 vs. control t = 2937, df = 13, Student’s *t*-test unpaired two-tailed. N = 7 to 8 in each group.

Unlike microglial cells, the density of GFAP^+^ cells was significantly reduced (14%; *p* = 0.011) in the SNc of animals treated with CBD, as shown in Figure 5A–C. Nevertheless, the treatment with CBD did not affect the phenotype of these cells. Indeed, no differences in the number of GFAP^+^ cell polarized towards the cytotoxic A1 (GFAP^+^/CD32^+^) or cytoprotective A2 (GFAP^+^/CD206^+^) phenotype were observed in animals treated with CBD compared to controls (Figure 5B–D).

### 2.3. Cannabidiol Induces the Activation of TRPV1-CNTF Cascade in the Astrocytes

**Figure 6:**
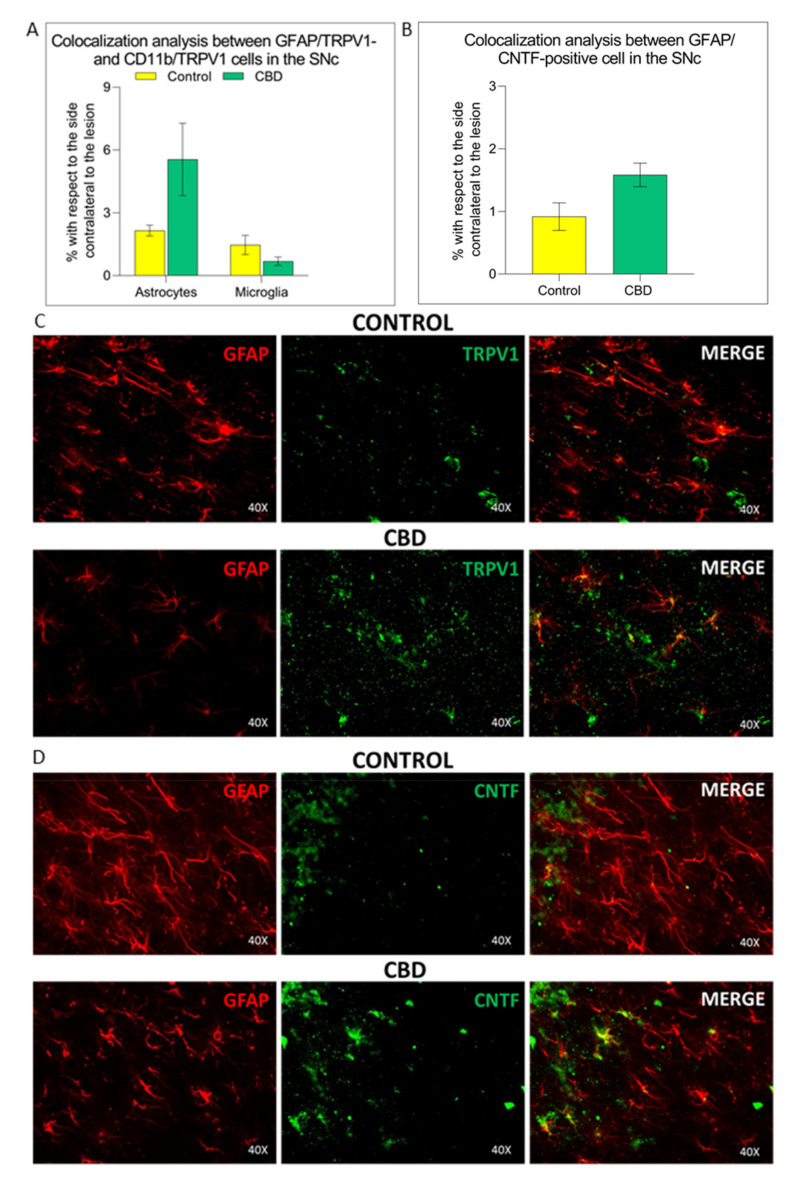
TRPV1 and CNTF glial expression in the substantia nigra. (**A**) Quantification of TRPV1 expression in astrocytes and mi-croglia. (**B**) Quantification of CNTF expression in astrocytes. (**C**,**D**) Representative images of the TRPV1 and CNTF expression in both glial subpopulations in the SNc, in both experimental groups. The results are expressed as mean ± SEM. N = 4 to 5 in each group.

**Figure 7:**
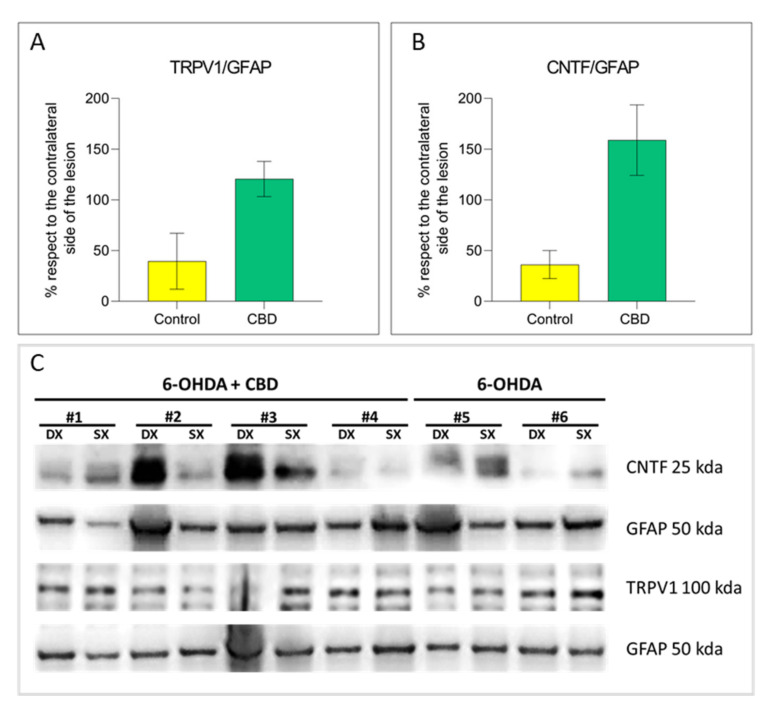
TRPV1 and CNTF astrocytic expression in the substantia nigra. (**A**) Quantification of TRPV1 expression in astrocytes. (**B**) Quantification of CNTF expression in astrocytes. (**C**,**D**) Representative images of Western Blot for TRPV1, CNTF and GFAP expression in the lesioned (Dx) and unlesioned (Sx) SNc, in both experimental groups. Results are expressed as mean ± SEM. N = 2 to 4 in each group.

In order to scrutinize the potential molecular mechanisms underlying CBD modulation of astrocytes, we focused our attention on TRPV1 activation, being CBD a TRPV1 agonist [19]. In CBD-treated animals, increased expression levels of TRPV1 receptor were observed by both immunohistochemical analysis and Western Blot in astrocytic cells compared with the control group (IHF: 61%, *p* = 0.063; WB: 81%, *p* = 0.059). In contrast, no differences in TRPV1 receptor expression levels on microglial cells between two experimental groups were found (Figure 6A–C and Figure 7A). Colocalization analysis and Western Blot showed that the increased astrocytic TRPV1 expression in CBD-treated animals is accompanied by a rise in the levels of the ciliary neurotrophic factor (CNTF) compared to control animals, with a difference bordering on statistical significance (IHF: 67%, *p* = 0.052; WB: 122%, *p* = 0.080) (Figure 6B,C and Figure 7B).

## 3. Discussion

Current pharmacological treatments of PD are essentially focused on alleviating the characteristic motor symptoms by compensating the loss of dopamine in the nigrostriatal pathway, without affecting the progression of the disease. Therefore, the identification of new therapeutic strategies capable of slowing down or counteracting the neurodegenerative process that characterizes PD is one of the major challenges in this field. Natural compounds, especially phytocannabinoids, represent potential candidates in the treatment of neurodegenerative diseases. In particular, CBD, one of over 100 phytocannabinoids identified in Cannabis sativa, shows anti-inflammatory and antioxidant actions, which make it a potential and promising candidate in the PD context [15,26,27,28,29,30]. In this study, we evaluated the neuroprotective and symptomatic efficacy of chronic treatment with CBD in a rat model of PD, based on the unilateral intrastriatal infusion of 6-OHDA. Animals treated with CBD showed a significant reduction of the striatal terminal degeneration and cell body loss in the SNc in comparison with the control group. Interestingly, the increased survival of dopaminergic neurons was accompanied by a recovery of motor performance. The results of this study agree with previous findings, further supporting the neuroprotective and symptomatic action of CBD treatment in different in vivo models of neurodegeneration [7,31,32,33].

In contrast to pre-clinical studies, the results on motor symptoms obtained in clinical trials are not as straightforward. While CBD treatment exhibited beneficial effect on most non-motor symptoms of PD patients, such as psychosis and sleep disorders, it does not show any notable efficacy on motor symptoms [34,35,36]. This discrepancy could be due to the dosages of CBD, which are lower than the one chosen in the present study. This hypothesis is supported by the results of a recent clinical study, in which PD patients are treated with escalating doses of CBD until the target dose of 20 mg/kg/day (per os), which is much higher than doses used in previous studies [37]. In particular, a beneficial effect was observed on tremor, as assessed by the reduction of the Movement Disorder Society-Unified Parkinson’s Disease Rating Scale total and motor scores, nocturnal sleep, and emotional and behavioral dyscontrol [37]. Another factor to be considered is the administration route, which also differs between pre-clinical and clinical studies. In the exploratory, as well as in preclinical studies, administration routes that provide higher bioavailability of the drug—such as intraperitoneal and intravenous route- are commonly used to prove the efficacy of a drug [38]. Therefore, the oral administration at relatively low doses used in the abovementioned clinical trials may have reduced the bioavailability of CBD and possibly some of its effects. It is noteworthy that the choice of pharmacological administration by intraperitoneal injection adopted in the present study was based on the properties of purified CBD (crystallised form) since this highly lipophilic form made the oral administration impossible. Moreover, based on previous pre-clinical studies [7,31,39] we employed a dosage which demonstrated beneficial effects in combination with a good tolerability profile. Last, we cannot exclude that the symptomatic improvement, observed in our study, may be attributed to starting the treatment shortly after the lesion induction, while the current clinical studies administered CBD after a diagnosis is made (i.e., about 50% of dopaminergic neurons in the brain are already destroyed [40]). Although this could represent a limitation of our study, this treatment paradigm was essential to explore the neuroprotective effects of this drug. In addition, chronic CBD treatment led to a decrease of the number of reactive astrocytes in the SNc without affecting microglial activation, as demonstrated by the comparable number of CD11b positive microglial cells in animals treated with CBD and controls. However, it should be noted that CBD-treated animals show an increase in the number of microglia endpoints and branch length, an index of the resting state of these cells. Indeed, under physiological conditions, microglia cells are characterized by extensive and branched processes whereas, under pathological conditions, these cells undergo morpho logical changes characterized by a reduction in both the number and the length of its ramifications [21,22]. Therefore, CBD did not reduce the extent of microglial response in the SNc, but it induced a moderate restoration of the resting condition in these cells. No straightforward phenotypic variations towards the cytotoxic or cytoprotective phenotype were instead observed after CBD treatment in both glia cell populations. These results confirm previous studies showing an immunomodulatory effect of CBD on glial cells [41,42,43,44,45], characterized by the reduction in the number of active cells and in the consequent release of pro-inflammatory factors, such as TNFα, COX-2 and iNOS [33,46]. These effects could account for the therapeutic action exerted by CBD in this study.

In order to further investigate the potential molecular mechanisms underlying the action of CBD, the expression levels of TRPV1 vanilloid receptor in the SNc were evaluated. Indeed, together with its antagonistic activity on cannabinoid CB1 and CB2 receptors [47], CBD can act as an agonist of the TRPV1 vanilloid receptor, playing a key role in the activation of anti-inflammatory response [19,48,49,50]. The assessment of the expression levels of TRPV1 in glial cells in the SNc highlighted a notably increased expression of the TRPV1 receptor in astrocytes of CBD-treated animals, but not in microglial cells. CBD-mediated activation of TRPV1 in the astroglial cells was accompanied by a marked increase in CNTF levels compared with the control group. Nam and collaborators (2015) showed that the activation of the TRPV1 astrocyte receptor is responsible for the neuroprotective and symptomatic effects observed in an animal model of PD based on MPP^+^ administration. In particular, capsaicin-induced activation of the TRPV1 receptor on the astrocytes enhances the endogenous neuroprotective response promoted by these glial cells, through the production and release of the ciliary neurotrophic factor (CNTF) [20].CNTF is a member of the interleukin-6 (IL-6) cytokine family that is almost exclusively expressed in the nervous system [51] where it is released by astrocytes, which increase its production following brain injury [20,21,52,53]. In addition to promoting adult neurogenesis [52], CNTF showed to increase survival of neurons after injury [52,54] and improve cognitive and memory function in rodent models [39]. As CNTF receptor expression in the SNc dopaminergic neurons is known [20,55], and according our preliminary results, we hypothesize that CNTF released by the astrocytes after CBD-induced TRPV1 activation might promote the survival of dopaminergic neurons, resulting in a recovery of motor performance as shown by recent studies [20,21,56]. Future studies will be required to validate the involvement of this pathway as one of the potential mechanisms of action of CBD in this animal model. In conclusion, the present study demonstrated the neuroprotective, anti-inflammatory and symptomatic effects of CBD treatment in an animal model of PD, potentially via the activation of astrocytic TRPV1-CNTF pathway. Although it cannot be excluded that other signaling pathways can contribute to the abovementioned effects—according to the pleiotropic action of CBD—the results of this study overall support the therapeutic potential of this phytocannabinoid as disease-modifying and symptomatic treatment for PD.

## 4. Materials and Methods

### 4.1. Animals

Male Sprague–Dawley rats (Charles River, Calco, LC, Italy) were anaesthetized with Sodium thiopental (50 mg/kg, i.p.) and placed in a stereotaxic frame (Stoelting, Chicago, IL, USA). They received a unilateral injection of 6-OHDA (20 μg/3 μL in saline/0.02% ascorbic acid; Sigma, St. Louis, MO, USA) into the right striatum (1.0 mm anterior, 3.0 mm lateral and 5.0 mm ventral, with respect to bregma and dura). All animal experiments were carried out with strict observance of protocols and guidelines approved by Local Committee, Italian Minister of Health and European Union legislation (Permit Number: 7/2019-PR of 8 January 2019 in compliance with article 31, D.Lgs.n. 26/2014).

### 4.2. Drug and Study Design

CBD (Purity: 99.5% (HPLC)—Linnea SA, Riazzino, TI, Switzerland) was prepared by isolation and purification from *Cannabis sativa* L. aerial parts. Prolonged decarboxylation allowed the conversion of the natural acidic form cannabidiolic acid to CBD. The procedure required refining steps with final crystallization. The treatment of rats (*n* = 12/group) with CBD (10 mg/kg/day, in Tween 80-saline 1:16, i.p.) or vehicle (control group) started 24 h after surgery (6-OHDA injection) and lasted 28 days. The day before neurotoxin infusion, the baseline motor performance was evaluated by cylinder and rotarod test and the tests were repeated before the sacrifice, the apomorphine-induced rotational behavior was assessed only before the sacrifice. Animals were deeply anesthetized with Sodium thiopental (150 mg/kg, i.p.) and transcardially perfused with saline and ice-cold 4% paraformaldehyde (Merck, Darmstadt, Germany). Brains were rapidly removed, post-fixed for 24 h in the same fixative and subsequently transferred in solutions of sucrose at increasing concentrations (up to 30%). Brains were then cut in serial coronal sections (40 μm) containing both the striatum and the substantia nigra pars compacta (SNc) using a microtome (Histo-line Laboratories) and underwent immunohistochemical staining.

### 4.3. Behavioral Evaluation

All tests were performed during the light phase (10:00–16:00), in full compliance with the directive of the European community. Cylinder test: Rats were placed individually in a glass cylinder (21 cm diameter, 34 cm height) for 5 min, and their behavior was recorded by video camera. The number of wall contacts made by the rat with the left or right forepaw was counted and preference of paw use was calculated using the following formula: (Ipsilateral/(ipsilateral + contralateral) − (contralateral/ipsilateral + contralateral)). Apomorphine-induced rotational test: The animals were injected with apomorphine (0.5 mg/kg dissolved in saline with 0.2% ascorbic acid, i.p.). The procedure induces a rotational motor response toward the opposite side to that of the 6-OHDA induced lesion. This response was evaluated using an automatic rotameter connected to the rat by means of an plastic belt for 30 min (Bioseb, Largo, FL, USA) and calculated by subtracting the total number of ipsilateral rotations from the total number of contralateral rotations. Rotarod test: Animals were briefly pre-trained on an automated 4-lane rotarod unit (Panlab, Spain) using both fixed and accelerating speed protocols, as reported in a previous study [57]. For the constant speed protocol, rats were placed on the rod and sequentially tested at 12 rpm for a maximum of 120 s. The animals were tested three times with a 10-min resting interval between each trial. For the incremental speed protocol, rats were placed on a rod that accelerates smoothly from 4 to 20 rpm over a period of 3 min. For both protocols, the time spent on the rod was recorded automatically for each animal.

### 4.4. Immunohistochemical Staining

The nigrostriatal lesion was assessed by immunohistochemistry directed against the dopaminergic marker tyrosine hydroxylase (TH) on coronal sections of both SNc and striatum. Briefly, sections were processed with a rabbit anti-TH primary antibody (1:2000, Chemicon AB152) and a biotinylated anti-rabbit immunoglobulin G secondary antibody (1:500, Vector Laboratories, San Francisco Bay Area, CA, USA) and revealed using a commercial kit based on the avidin-biotin technique (Vectastain ABC Elite kit, Vector Laboratories). Reaction products were developed using nickel-intensified 30-30-diaminobenzedine tetrahydrochloride for 1 min (DAB Substrate Kit for Peroxidase, Vector Laboratories). Microglia and astrocyte activation and polarization in the SNc was assessed by triple immunofluorescent staining directed against (a) the Cluster of Differentiation molecule 11 b (CD11b) (1:300, Serotec MCA275 R) for microglia or the Glial Fibrillary Acidic Protein (GFAP; 1:1000, Sigma, USA, G3893) for astrocytes, (b) the Cluster of Differentiation molecule 32 (CD32, 1:300, Santa Cruz, Dallas, TX, USA, sc-28842) or CD206 (1:300, Santa Cruz, USA, sc-48758) for assessing cytotoxic (M1-A1) or neuroprotective (M2-A2) phenotypes, respectively) TH for SNc localization (1:200, Novusbio NB300-110). Transient Receptor Potential Vanilloid 1 (TRPV1) (1:1000, Alomone Labs, Israel, ACC-030) and ciliary neurotrophic factor (CNTF) (1:500, Millipore, Burlington, MA, USA, MAB338) levels in glial cells were assessed by double immunofluorescent staining. Alexa Fluor 350 (1:150, Thermo Fisher, Dallas, TX, USA), 488 and 594 (1:300, Thermo Fisher, Waltham, MA, USA) were used as secondary antibodies.

### 4.5. Image Analysis

Image analysis was performed using an AxioSkop2 microscope, equipped with Apotome 2, connected to a computerized image analysis system (AxioCam MR5, Zeiss, Gina, Germany) with dedicated software (AxioVision Rel 4.2, Zeiss, Germany). The striatal degeneration was expressed as the percentage of striatal volume deprived of TH immunoreactivity, with respect to the entire striatal volume. The number of TH-positive neurons in the SNc was counted bilaterally on every four sections throughout the entire nucleus by unbiased stereology using the optical fractionator method (Stereo Investigator System 9.03.2, Microbrightfield Inc., Williston, VT, USA). The results were expressed as the percentage of TH-positive neurons in the lesioned SNc compared with the intact hemisphere. Cell count of microglia and astrocytes was performed by analyzing three different SNc sections, chosen according to rostrocaudal coordinates. Cell density was assessed by counting CD11b- or GFAP-positive cells from a stack of 16 pictures (in a 0.04 mm [2] frame, 1 mm-thick, 40× magnification) taken from three discrete areas of the same SNc section. The analysis of microglia or astrocytes polarization was performed by evaluating the percentage of CD32-positive (M1-A1) and CD206-positive cells (M2-A2) of the total microglia cells or astrocytes. The microglia process length and number of endpoints were quantified using skeleton plugin in FIJI (NIH, Bethesda, MD, USA) [58]. The results obtained were expressed as a percentage of branch length (μm)/cell and number of endpoints/cell in the lesioned SNc compared with the intact hemisphere. Colocalization analyses for TRPV1 and CNTF in glial cells were accomplished by using the EzColocalization plugin in FIJI [59]. Metric matrix used in this analysis was the threshold overlap score (TOS).

### 4.6. Western Blot

Animals were sacrificed by decapitation and SNc area was rapidly removed and frozen on dry ice, and stored at −80 °C. Protein lysate was obtained by re-suspending SNc in ice-cold lysis buffer (CelLytic, Sigma, USA) containing diluted phosphatase (1:10, Roche, Monza, Italy) and protease inhibitors (1:25, Roche, Italy). After centrifugation, the supernatant was collected and protein concentration was measured using a Bicinchoninic Acid Protein Assay (Sigma, USA). Protein lysates were run on 10% gels, transferred onto nitrocellulose membranes (Biorad, USA) and western blot was performed. Membranes were blocked (Odyssey blocking buffer, LiCor, USA) and incubated overnight with the following primary antibodies: anti-GFAP (1:2000, Sigma, USA, G3893), anti-TRPV1 (1:500, Santa Cruz, USA); anti-CNTF (1:500, Millipore, Germany, MAB338). As secondary antibodies, anti-mouse 1:10000 secondary IgG HRP-Conjugated were used. Image analysis of western blots was performed using Azure 600 azure biosystems and software (LiCor, Biosciences, Rockville, MD, USA) and signal was normalized with the corresponding GFAP signal.

### 4.7. Statistical Analysis

The results are expressed as mean ± SEM. Statistical analysis was performed using GraphPad Prism 8 (GraphPad software, San Diego, CA, USA). Comparisons between groups were made using Student’s *t*-test unpaired two-tailed. Statistical significance was set at *p* < 0.05.

## Data Availability

The data that support the findings of this study are available from Zenodo, doi:10.5281/zenodo.5148268.
